# MicroRNA in T-Cell Development and T-Cell Mediated Acute Graft-Versus-Host Disease

**DOI:** 10.3389/fimmu.2018.00992

**Published:** 2018-05-07

**Authors:** Christian Koenecke, Andreas Krueger

**Affiliations:** ^1^Clinic for Hematology, Hemostasis, Oncology and Stem Cell Transplantation, Hannover Medical School, Hannover, Germany; ^2^Institute of Immunology, Hannover Medical School, Hannover, Germany; ^3^Institute for Molecular Medicine, Goethe-University Frankfurt, Frankfurt am Main, Germany

**Keywords:** T cell, microRNA, graft-versus-host disease, graft-versus-tumor effect, differentiation

## Abstract

Acute graft-versus-host disease (GvHD) is still a major cause of treatment-related mortality after allogeneic stem cell transplantation. Allo-antigen recognition of donor T cells after transplantation account for the onset and persistence of this disease. MicroRNAs (miRNAs) are molecular regulators involved in numerous processes during T-cell development, homeostasis, and activation. Thus, miRNAs also contribute to pathological T-cell function during GvHD. Given their capacity of fine-tuning T-cell function, miRNAs have emerged as promising therapeutic targets to curtail acute GvHD, but simultaneously maintain T-cell-mediated graft-versus-tumor effects. Here, we review the role of key miRNAs contributing to the pathophysiology of GvHD. We focus on those miRNAs acting in T cells and for which a role in GvHD has been established in preclinical models. Finally, we provide an outlook for clinical application of this new therapeutic target for GvHD prevention and treatment.

## Introduction

Acute graft-versus-host disease (GvHD) is still a major complication after allogeneic hematopoietic stem cell or bone marrow transplantation (alloSCT). Allogeneic donor T cells are the main inducers of this too often lethal disease. Donor T cells recognize and respond to allo-antigen, use chemokine receptors and integrins to home to epithelial organs, such as the liver, the intestine, or the skin and orchestrate a significant immune response, which frequently results in severe organ damage. Unspecific prophylactic and therapeutic immunosuppressive treatment is the current clinical practice to improve patients’ outcome. However, the repertory of therapeutic and preventive options is limited and, therefore, new targets to control acute GvHD are urgently needed (1).

Since T cells orchestrate this disease, they are first choice to be targeted. Only naïve donor CD4 and CD8 T cells respond to allo-antigen *via* the T-cell receptor (TCR) in alloSCT, whereas central and/or memory T cells are not able to induce acute GvHD (2). Depending on several signals from the microenvironment, naïve T cells respond to allo-antigens in different ways. TCR signals are usually provided by antigen-presenting cells (APCs) *via* MHC class I or II molecules to CD8 and CD4 T cells, respectively. Recipient and donor APCs have differential impact on GvHD-induction by donor T cells (3–9). Furthermore, additional signals *via* cytokines are provided by the inflamed microenvironment and lead to onset and/or acceleration of this immune response (10). Whereas the plasticity of donor CD8 T cells seem to be limited, CD4 T cells develop into different subtypes during activation. T helper (Th) subtypes, such as Th1, Th2, Th17, and regulatory T cells (Treg) have distinct functions in the course of GvHD. The main drivers of acute GvHD, at least in rodents, are Th1 and Th17 cells (11–14). The cytokine release of such subtypes ultimately leads to tissue damage, which defines the clinical outcome of the disease. However, Th2 responses with cytokines such as IL-4, IL-5, IL-9, and IL-13 contribute to acute GvHD as well (15–17). It is believed that the impact on the pathophysiology of such cytokines depends on the timing and location of cytokines released by CD4 subsets. This is especially true for the Th1 cytokine IFN-γ, which is involved in inflammatory processes but can also facilitate immunosuppressive effects (18, 19). Further Th1 type cytokines TNFα and IL-2 have been tested for the prevention and treatment of GvHD not only in experimental models but also in patients with heterogeneous results (20). Th17 cells produce cytokines such as IL-17A, IL-17F, and IL-22 under the influence of IL-23 (21). A role for Th17 and associated cytokines such as IL-17A and IL-22 during acute GvHD has been shown, however, with controversial results. In one study, IL-17A deficiency leads to disease reduction (22), whereas another study shows that absence of Th17 cells exacerbates acute GvHD (23). IL-22 has been shown to be protective during GvHD by protection of recipients’ intestinal stem cells (24). A critical role in the pathophysiology of acute GvHD is attributed to Treg cells (25–27). It has been demonstrated in preclinical animal models that thymic-derived CD4^+^CD25^+^ natural Treg cells prevent the development of severe acute GvHD while preserving graft-versus-tumor (GvT) effects (28). Clinical studies are currently underway to test the therapeutic potential of natural Treg cells as a cellular therapy (29). However, the role of induced Treg cells in the context of GvHD is less clear (30), and it is controversially discussed whether such cells are suitable for therapeutic usage. Other CD4 T cell subsets, such as T follicular helper (Tfh) cells seem to have a role in chronic GvHD, but not acute GvHD (31). Furthermore, there is some evidence that also NK cells, natural killer T cell and invariant natural killer (iNK) T cells contribute to acute GvHD pathophysiology (25).

## MicroRNAs (miRNAs) Controlling T-Cell Development and Function

MicroRNAs act as post-transcriptional regulators predominantly by facilitating mRNA degradation or inhibiting translation. For most miRNAs, multiple, even hundreds, of target mRNAs have been predicted *in silico*, and many of those have been validated experimentally at least in some cell types (32). Conversely, mRNAs frequently contain binding sites for multiple miRNAs. Thus, miRNAs are likely to play diverse roles in controlling gene expression in different contexts dependent on both miRNA and mRNA concentration and their binding affinity. Of note, many mRNAs display a limited degree of repression through the action of an individual miRNA (33, 34). Accordingly, a major function of miRNAs might lie in controlling noise in protein expression of lowly expressed genes (35, 36) or in generating expression switches (37). In addition, it has been suggested that individual miRNAs co-target multiple mRNAs within the same pathway, thus being able to functionally control biological processes despite low levels of repression of individual mRNAs (38). In turn, some mRNAs appear to be key targets of a wide variety of miRNAs. One example constitutes the negative regulator of PI3K signaling, Pten. In addition, a pseudogene homologous to Pten has been described to act as negative regulator of miRNA-mediated regulation of Pten *via* competition for miRNA binding (39–41). Although the hypothesis that miRNA function is regulated *via* the abundance of corresponding miRNA-binding sites in competing mRNAs is compelling, quantitative analysis of miRNA copies and abundance of miRNA response elements suggested that individual competing RNAs are unlikely to significantly contribute to target derepression (42–45). Recently, Heissmeyer and colleagues demonstrated that the RNA binding Protein Roquin blocks miRNA-mediated regulation by occupying a binding site for miR-17–92 in the 3′ untranslated region (UTR) of Pten mRNA, thus adding another level of complexity to the system (46).

Despite the described complexity in miRNA–mRNA interdependence, functionally relevant regulatory one miRNA—one mRNA relationships have been demonstrated using targeted deletion of defined miRNA-binding sites in individual genes. For instance, some, but not all, functions of miR-155 in the immune system could be ascribed to its repression of Socs-1 (47). On the other hand, targeted deletion of a miR-142-binding site in Cdkn1b did not phenocopy aberrant proliferation of thymocytes observed in miR-142-deficient mice (48).

Unsurprisingly, miRNAs also play a fundamental role in T-cell development and differentiation. Thus, T-lineage specific ablation of genes essential for processing of the vast majority of miRNAs, such as Dicer, Drosha, or Dgcr8 results in aberrant T-cell development and function. Loss of virtually all miRNAs early during T-cell development results in increased levels of apoptosis of thymocytes and, concomitantly, reduced thymocyte numbers (49, 50). Deletion of Dicer at later developmental stages showed that miRNAs are critical for maintenance of peripheral T cells, most notably, the CD8 T cell compartment (51). Within the CD4 T cell compartment, loss of virtually all miRNAs resulted in a bias toward a Th1 differentiation program and against differentiation toward the Th2 and Th17 lineages (51, 52). In addition, generation of Tfh cells is dependent on miRNA (53). Furthermore, miRNAs are critical for intrathymic development as well as peripheral induction of Treg cells (54). Treg-cell specific deletion of all miRNAs resulted in fatal autoimmunity due to defective peripheral Treg-cell homeostasis (55–57).

**Figure 1:**
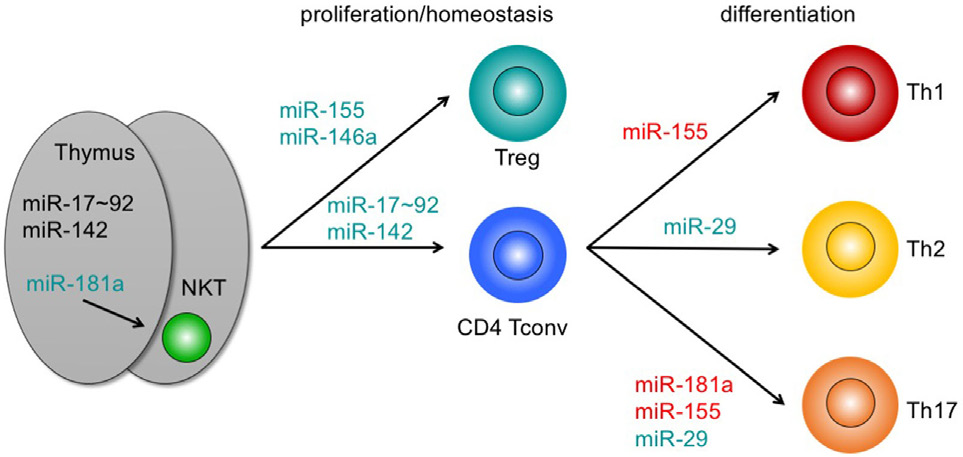
Pathophysiologically relevant microRNAs (miRNAs) in graft-versus-host disease with a functional role in T-cell development, homeostasis, and differentiation. miRNAs adjacent to arrows indicate those miRNAs that positively contribute to intrathymic development (left), proliferation and/or homeostasis (center), or differentiation of T cell subsets. miRNAs highlighted in green mirror exert major functions. Loss of these miRNAs partially mirrors depletion of all miRNAs in mice. miRNAs highlighted in red indicate miRNAs with non-dominant functions. For details, see text. Abbreviations: NKT, natural killer T cell; Treg, regulatory T cell; Tconv, conventional T cell; Th, T helper cell.

In both T-cell development and differentiation, individual miRNAs have been extensively characterized [reviewed in Ref. (58–60)]. Here, we focus mainly on describing physiological roles of miRNAs that have been directly linked to GvHD in preclinical models or may affect critical T-cell function in the context of GvHD (Figure 1; Table 1). By comparing consequences of deletion of individual miRNAs versus deletion of the total pool of miRNAs, Dooley and colleagues identified those individual miRNAs as dominant, deletion of which helps to explain the consequences of loss of the complete miRNAome (59). In contrast, loss of individual miRNAs that does not mirror complete miRNA deficiency rather indicates functions in line with the role of miRNAs to fine-tune biological processes.

**Table 1:** MicroRNAs (miRNAs) acting in T cells, for which a role in graft-versus-host disease (GvHD) has been established in preclinical models.

miRNA	Physiologic function	Role in GvHD	Reference
miR-17–92	Promotes early T-cell development Promotes activation-dependent T-cell proliferation Regulates regulatory T cell (Treg) cell suppressive function	Pathogenic	(60–65)
miR-142	Curtails T-cell progenitor numbers Promotes T-cell proliferation	Pathogenic	(47, 66)
miR-146a	Promotes Treg-cell homeostasis	Beneficial	(67–69)
miR-146b	n.d.	Pathogenic	(70)
miR-153-3p	Prevents immunosuppression through indoleamine-2,3-dioxygenase	Pathogenic	(71, 72)
miR-155	Promotes Treg cell homeostasis	Pathogenic	(73, 74)
miR-181a	Rheostat for T-cell receptor signaling Promotes development of invariant natural killer T cells	Beneficial	(75–84)

A cluster of six related miRNAs, miR-17–92, confers competitive fitness to the earliest T-cell progenitors in the thymus and pre-thymic progenitors by regulating IL-7 receptor levels and IL-7 signaling (85). Conversely, miR-142 curtails numbers of early T-lineage progenitors, although loss of this miRNA inhibits proliferation and ultimately results in peripheral lymphopenia (48). Together, miR-17–92 and miR-142 help to explain the majority of T-lineage developmental defects observed upon early depletion of all miRNAs. Members of the miR-181 family are among the most prominently expressed miRNA species in thymocytes preceding and/or undergoing selection (75, 86). miR-181a targets a group of phosphatases, all of which act as negative regulators of TCR signaling (76). Accordingly, miR-181a sensitizes cells toward TCR signaling and may serve as a rheostat during positive selection (76,  77). Conversely, loss of miR-181a-1 and miR-181b-1 dampens TCR signaling in double-positive thymocytes (78, 79). Nevertheless, the effect of loss of miR-181a/b-1 on selection of conventional T cells (Tconv) is comparatively mild. In an HY-TCR transgenic model, loss of miR-181a/b-1 results in an increase in positively selected TCR-transgenic T cells (79). In contrast, miR-181a/b-1 is critical for development of agonist-selected iNK T cells (78, 80, 81). Although control of agonist-selected populations by miR-181 can be plausibly explained by regulation of TCR signal strength and elevated TCR expression was able to completely restore iNKT cell development, alternative mechanisms of actions, such as *via* regulation of Pten, cannot be fully ruled out (66, 78, 80).

Once outside the thymus miRNAs are critical for peripheral maintenance and proliferation of T cells. Thus, T cells deficient in miR-142 fail to expand after egress from the thymus and do not efficiently proliferate upon TCR triggering (48, 61). Similarly, T cells lacking miR-17–92 proliferate inefficiently after stimulation *in vitro* and after infection, a defect that can be partially restored by re-introduction of miR-17 and miR-92a (62, 63, 82). Despite its low levels of expression when compared to thymocytes, miR-181a has been proposed to regulate responsiveness in peripheral CD4 T cells, especially in humans. Notably, expression of miR-181a progressively declines with age, resulting high sensitivity toward TCR signals in cord blood T cells and impaired sensitivity in naive T cells from aged individuals (83, 87).

Loss of miR-29 results in a bias toward Th1 differentiation similar to that observed in pan-miRNA-deficient T cells *via* derepression of key transcription factors T-bet and Eomes as well as IFN-γ (62, 67). restoration of miR-29 expression in these mice can partially overcome this defect (62). miR-146a and miR-155 constitute critical miRNAs for function and homeostasis of Treg cells. Loss of miR-146a derepressed transcription factor Stat1 and, consequently, resulted in dysregulated expression of IFN-γ and accompanying immunopathology (73). The Foxp3-dependent miR-155 promotes Treg-cell homeostasis by controlling responsiveness to IL-2 signals *via* repression of Socs-1 (64). Combined these data place Socs-1 in a central miRNA-controlled hub of Treg cell function, because both effector signals, such as Stat1/IFN-γ as well as homeostatic signals *via* Stat5/IL-2 are regulated by Socs-1. Of note, disruption of the miR-155/Socs-1 axis by introducing mutations into miR-155-binding sites in the *Socs1* 3′UTR perturbed Treg-cell homeostasis under competitive conditions, but not at steady state, highlighting the complexity of miRNA-dependent gene regulation (47). Interestingly, although miR-17–92 contributes to regulating suppressive function *via* regulating production of IL-10, this cluster does not control proliferation or survival of Treg cells (88). Specific induction of miR-181a in differentiating Th17 cells can selectively sensitize these cells toward TCR-mediated signals, thus contributing to controlled memory T cell formation (89).

## miRNA Function in T Cells in the Context of Acute GvHD

For a number of miRNAs, a role in the pathophysiology of acute GvHD has been described. miR-155 is important for Treg cell homeostasis and was also linked to differentiation of Tconv in autoimmune responses such as in experimental autoimmune encephalomyelitis (74, 90). miR-155 expression is upregulated in donor T cells in an experimental GvHD model and also in intestinal tissue of GvHD patients. Furthermore, donor T cells lacking miR-155 induce reduced GvHD, whereas miR-155 overexpression accelerates the course of the disease in experimental alloSCT. Consistently, blocking miR-155 by locked-nucleic-acid-modified (LNA−) anti-miR-155 oligonucelotides diminishes GvHD. In addition to alterations in T cell differentiation, impaired homing to GvHD target organs due to decreased expression of homing receptors CCR5, CXCR4, and S1P1 on effector T cells, has been proposed as the underlying molecular mechanism of blocking miR-155 (65).

Since miR-142 and miR-17–92 constitute important mediators of T cell proliferation, their contribution to GvHD pathophysiology was not unexpected. Donor T cells deficient for miR-142 show impaired cell cycle progression and simultaneously reduced proliferation, which in turn leads to reduced GvHD severity and mortality in multiple experimental alloSCT models. Mir-142 deficiency in T cells leads to upregulation of atypical E2F transcription factors E2f7 and E2f8, which are both known targets of this miRNA. Thus, targeting the interaction between miR-142 and E2F transcription factors, might serve as a potential therapeutic approach (61). T cells deficient in miR-17–92 proliferate inefficiently after stimulation *in vitro* and after infection (63, 82). In line with these data, miR-17–92-deficient donor T cells were unable to induce lethal acute GvHD, which could be mostly ascribed to miR-17 and miR-19b (68). Interestingly, in this model, the GvT effect was preserved, which might be due to the fact that proliferation, survival, and cytotoxic functions of CD8 T cells are at least partially maintained in the context of GvHD, when miR-17–92 is targeted. These data highlight the capacity even of broadly acting miRNAs to fine tune T cell function in a context-dependent manner.

The miR-146 family includes two members, miR-146a and miR-146b, which are both involved in acute GvHD pathophysiology. miR-146a overexpression in donor T cells reduces GvHD and in turn lack of this miRNA leads to increased GvHD severity with high levels of TNFα. In line with these experimental data, the occurrence of the SNP rs2910164 in alloSCT donors, which reduces miR-146a expression, is associated with higher incidence of severe GvHD in a patient cohort (69). One target of miR-146a in T cells is the TNF-receptor-associated factor 6 (TRAF6) (70). TRAF6 regulates transcription of TNFα *via* NF-κB and high levels of miR-146a reduces TNFα transcription in donor T cells and thereby reduces GvHD. TRAF6 is also a target of miR-146b and antagomir-mediated knockdown of miR-146b leads to enhanced TRAF6 expression. Increased TRAF6, in turn, activates NF-κB and leads to enhanced survival, proliferation, and suppressive activity of natural Treg cells. Hence, miR-146b antagomir-treated human Treg cells decrease mortality in a xenogenic GvHD-model and might, therefore, improve the adoptive Treg cell therapy (91). The opposing functions of the closely related miR-146a and miR-146b shed light on the complexity of miRNA-based intervention to treat GvHD.

Indoleamine-2,3-dioxygenase (IDO) is a critical enzyme in providing essential amino acids for T cell proliferation and exerts immunosuppressive effects. Increased levels of IDO levels prevent acute GvHD (72). IDO is directly targeted by miR-153-3p and miR-153-3p. Antagomir for miR-153-3p and miR-153-3p led to higher IDO expression in experimental GvHD and delayed the course of disease (84).

miR-181a acts as a rheostat for T cell sensitivity to antigen by downregulation of several phosphatases downstream of the TCR (76). Primary T cells overexpressing miR-181a failed to induce experimental GvHD, whereas, conversely, donor T cells lacking miR-181a/b-1 accelerated acute GvHD in the same model (92). Overexpression of miR-181a resulted in decreased T cell survival, most likely because of reduced expression of anti-apoptotic BCL-2 protein expression. Repression of BCL-2 protein in this context likely results from a combination of it being a direct target of miR-181a as well as a consequence of apoptosis regulation dependent on TCR signal strength. This study points toward BCL-2 inhibition as a novel therapeutic strategy in prevention of allo-reactivity and highlights miRNAs as titrable therapeutic targets in order to prevent GvHD while preserving GvT effects.

## Future Directions

Understanding the role of miRNAs in physiological T cell function and in the context of disease has the potential to open up new avenues for therapy. Most of this knowledge is derived from the analysis of rodent models and is only beginning to be complemented by studies in human primary T cells (93). To a large extent, miRNAs and their mRNA target sites are evolutionary conserved. However, some notable exceptions have been described. Thus, miR-125b controls human T cell differentiation *via* a number of mRNA targets lacking corresponding miR-125b binding sites in mouse, including the genes encoding IFN-γ and IL-10RA (93). In terms of therapeutic delivery, alloSCT has the distinct advantage that cells might already be manipulated prior to transplantation *ex vivo*. Recently, methods for efficient CRISPR/Cas9-mediated genome engineering of primary human T cells have been reported (94). In addition, highly potent chemically modified miRNA analogs or antagonists are beginning to emerge that can be directly delivered *in vivo* (95, 96), some of which have already been employed experimentally in rodent models of GvHD (65, 69). In order to prevent off-target effects in case of systemic delivery such miRNAs can be further modified to generate compounds that can be selectively activated through light in typical GvHD target organs, such as skin and gut (97).

Our knowledge of miRNA action is far from complete and a number of miRNAs with a role in T cell function, for instance, miR-148a, miR-182, and miR-326, may ultimately also play a role in GvHD (98–100). Similarly, a number of miRNAs have been identified as putative biomarkers for GvHD, such as miR-194, but a functional role in pathogenesis remains to be established (101). Finally, miRNAs also play a role in non-T cells during GvHD. For instance, miR-100 has been reported to limit neovascularization in the intestine and miR-155, which functions as a key regulator of Treg cells as well as Tconv, negatively impacts upon dendritic cell function during GvHD (102, 103).

## Conclusion

MicroRNAs play critical role in many biological processes including T-cell development and differentiation. Given that GvHD is a predominantly T-cell driven disease, miRNAs have emerged as attractive therapeutic targets for *ex vivo* and potentially even *in vivo* modulation. A better understanding of miRNAs controlling physiologic T-cell function has resulted in efficient translation of manipulation of miRNAs in preclinical models of GvHD. However, despite the generally broad interspecies conservation of miRNAs, translation into clinical treatment of GvHD remains a challenge.

## Author Contributions

All authors listed have made a substantial, direct, and intellectual contribution to the work and approved it for publication.

## Conflict of Interest Statement

The authors declare that the research was conducted in the absence of any commercial or financial relationships that could be construed as a potential conflict of interest.
